# Physical match demands of four LIQUI-MOLY Handball-Bundesliga teams from 2019–2022: effects of season, team, match outcome, playing position, and halftime

**DOI:** 10.3389/fspor.2023.1183881

**Published:** 2023-05-24

**Authors:** Christian Saal, Christian Baumgart, Florian Wegener, Nele Ackermann, Florian Sölter, Matthias W. Hoppe

**Affiliations:** ^1^ Movement and Training Science, Faculty of Sport Science, Leipzig University, Leipzig, Germany; ^2^ Department of Movement and Training Science, University of Wuppertal, Wuppertal, Germany; ^3^ Athletic Center, MT Melsungen, Melsungen, Germany

**Keywords:** LPS, IMU, team performance, handball, activity, tracking, metabolic power

## Abstract

**Introduction:**

Due to the development in team handball, there is a need to optimize the physical capacities of team handball players for which knowledge of the physical match demands is essential. The aim of this study was to investigate the physical match demands of four LIQUI-MOLY Handball-Bundesliga (HBL) teams across three seasons with respect to the effects of season, team, match outcome, playing position, and halftime.

**Methods:**

A fixed installed local positioning system (Kinexon) was used, collecting 2D positional and 3D inertial measurement unit data at 20 and 100 Hz, respectively. The physical match demands were operationalized by basic (e.g., distance, speed, and acceleration) and more advanced variables (e.g., jumps, throws, impacts, acceleration load, and metabolic power). A total of 347 matches (213 with an additional ball tracking) were analyzed from four teams (one top, two middle, and one lower ranked) during three consecutive seasons (2019–2022). One-way ANOVAs were calculated to estimate differences between more than two groups (e.g., season, team, match outcome, playing position). Mean differences between halftimes were estimated using Yuen’s test for paired samples.

**Results:**

Large effects were detected for the season ($0.6\leq \;\;\hat{\;\;\xi }\leq 0.86$), team ($0.56\leq \;\;\hat{\;\;\xi }\leq 0.72$), and playing position ($0.64\leq \;\;\hat{\;\;\xi }\leq 0.98$). Medium effects were found for match outcome ($\;\;\hat{\;\;\xi }\leq 0.36$) and halftime ($\;\;\hat{\;\;\xi }\leq 0.47$).

**Conclusion:**

For the first time, we provide a comprehensive analysis of physical match demands in handball players competing in the LIQUI-MOLY Handball-Bundesliga. We found that physical match demands differ on that top-level with up to large effect sizes concerning the season, team, match outcome, playing position, and halftime. Our outcomes can help practitioners and researchers to develop team and player profiles as well as to optimize talent identification, training, regeneration, prevention, and rehabilitation procedures.

## 1. Introduction

Team handball is a contact team-sport with the objective to score more goals than the opponent (1). The match performance is a multidimensional construct, including physical, technical-tactical, cognitive, and further factors. It is determined by the individual performance of the players and their interaction (2). In team handball, the physical match demands involve activities as running, jumping, throwing, and blocking, whereby playing positional differences exist (3). During the last decades, it is assumed that the physical match demands have increased in team handball (4). Potential reasons for that development may be related to more competition per seasons (2), decreased performance differences between teams (5), and rule changes (e.g., passive game, goal keeper substitution, and fast throw off following a goal) (4). Due to that development, there is a need to optimize the physical capacities of team handball players for which knowledge of the physical match demands is essential (1).

To quantify physical match demands in team handball, player tracking technologies, particularly local positioning systems (LPS) and inertial measurement units (IMU), have become standard procedures (6). In fact, since the season 2019/2020, all matches of the German LIQUI-MOLY Handball Bundesliga are systematically tracked by these technologies (Kinexon Web). Based on the collected data, it is possible to compute basic (e.g., distance, speed, and acceleration) and more advanced variables (e.g., jumps, throws, impacts, acceleration load, and metabolic power), as indicators of the physical match demands (7–9). Specifically, such data may be helpful to develop team and player profiles as well as to optimize talent identification, training, regeneration, prevention, and rehabilitation procedures (10, 11).

Previous studies show that running related match demands such as total distance, speed, and acceleration do not differ with respect to the match outcome in top-level team handball (12, 13). However, other studies found differences between winning and losing teams in high-intensity actions (14, 15). Also, differences between playing positions were comprehensively investigated in previous studies (9–12, 16), indicating specific profiles for wingers, centers, and pivots. Additionally, fatigue during certain match periods was studied (2, 3, 17, 18) and the findings reveal that high-intensity movements as the number of stops, change of directions, and also one-to-one situations (3) as well as distances covered at fast running and sprinting (17) decrease from first to second half and also towards the end of both halves. However, there is no study that has investigated the aforementioned aspects of the physical match demands across multiple seasons and for several top-level handball teams. Since most of the previous studies have investigated one tournament or few players, there is a need for a comprehensive study, systematically taking the physical match demands of top-level handball teams by LPS and IMU technologies into account.

Thus, the aim of this study was to investigate the physical match demands of four LIQUI-MOLY Handball-Bundesliga teams across three seasons with respect to the effects of season, team, match outcome, playing position, and halftime.

## 2. Methods

### 2.1. Study design and ethical aspects

Within a retrospective observational study, a range of common physical variables (7) (Table 1) of four men’s handball teams (A, B, C, and D) competing in the LIQUI-MOLY Handball-Bundesliga (HBL) were analyzed across the seasons 2019/20, 2020/21, and 2021/22. Since the season 2019/2020, all matches of the Handball-Bundesliga are tracked by the same LPS technology (Kinexon GmbH, Munich, Germany), which allowed valid comparisons. To investigate the effect of season, team, match outcome, playing position, and halftime, differences in means were analyzed. The match outcomes were obtained from the official HBL-LIQUI-MOLY website. The collected data is free available for each team competing in the HBL, and an approval was granted by the four clubs to analyze and publish the data. Therefore, Ethics Committee clearance was not required. All procedures were conducted in accordance with the Declaration of Helsinki.

**Table 1:** Description of used variables.

Variable	Unit	Description
Playing time	s	Time athlete spent on playing field (within field limits) during the match
Distance	m	Total distance covered by the player during the match based on the player’s LPS data
Max. speed	km/h	Highest speed value during sprint event. A sprint event is triggered when a player maintains a speed over a given speed threshold (15.12 km/h) during a minimum duration (0.5 s)
Time speed	s	Time spent in speed zones. The following five categories were used: very high: ≥22 km/h, high: 16 to 22 km/h, medium: 10 to 16 km/h, low: 4 to 10 km/h, very low: <4 km/h
Max. acceleration	m/s${}^{2}$	Maximum acceleration a player performed during the match. The value based on LPS data
Acceleration		Number of accelerations in zones. This metric counts the accelerations an athlete performs during the match. An acceleration event is detected if the athlete maintains an acceleration over an specific threshold (2 m/s${}^{2}$) over a minimum duration (0.5 s). The following four categories were used: very high: ≥3.5 m/s${}^{2}$, high: 3 to 3.5 m/s${}^{2}$, medium: 2.5 to 3 m/s${}^{2}$, low: <2.5 m/s${}^{2}$
Max. deceleration	m/s${}^{2}$	Maximum deceleration a player performed during the match. The value based on LPS data
Deceleration		Number of decelerations in zones. This metric counts the decelerations an athlete performs during the match. A deceleration event is detected if the athlete maintains a deceleration over an specific threshold (−1.5 m/s${}^{2}$) over a minimum duration (0.5 s). The following four categories were used: very high: ≥−3.5 m/s${}^{2}$, high: −3 to −3.5 m/s${}^{2}$, medium: −2.5 to −3 m/s${}^{2}$, low: <−2.5 m/s${}^{2}$
Max. metabolic power	W/kg	Highest value during the match. The metric metabolic power is an estimation of the energy that is required for a mechanical movement due to LPS data. It is calculated from the metrics speed and acceleration described in Osgnach et al. (19). It does not consider any exercise that does not produce a change of the position on the playing field (e.g. jumps, impacts, push-ups)
Time metabolic power	s	Time spent in metabolic power zones. The following four categories were used: very high: ≥25 W/kg, high: 15 to 25 W/kg, medium: 4 to 15 W/kg, low: <4 W/kg
Accumulated acceleration load	a.u.	The metric is calculated from IMU data and captures all movements in the X, Y, and Z axis (e.g. motion, jumps, and impacts) and is described in Boyd et al. (20). $\sum_{i=1}^{n}\sqrt{\frac{(a_{y1}-a_{y-1}{)}^{2}+(a_{x1}-a_{x-1}{)}^{2}+(a_{z1}-a_{z-1}{)}^{2}}{100}}$
Impacts		Number of collision events between players during match. This metric is threshold based and counts the events when two players collide. LPS as well as IMU data is used to detect impacts. To trigger an impact a big magnitude acceleration has to be registered from two players, who stand next to each other, at the same time
Jumps		This metric counts all jumps of a match. LPS as well as IMU data is used to detect jump events. To trigger jump event the athlete has to be in the air between 0.35 to 0.99 s
Passes		This metric counts all passes an athlete played during a match. The player’s and ball’s position data is used to detect the event
Shots		This metric counts the shots towards the goal of the match. The player’s and ball’s position data is used

Notes: a.u., arbitrary unit; Variables without units represents counts.

### 2.2. Sample

Table 2 shows number of players, played matches, and observations for each playing position of the four LIQUID-MOLY Handball Bundesliga teams across the three seasons. The teams finished the seasons accordingly: Team A: 1st to 3rd place; Team B: 7th to 8th place; Team C: 6th to 9th place; and Team D: 11th to 13th place. Thus, the teams can be considered as top (Team A), middle (Team B and Team C), and lower ranked (Team D).

**Table 2:** Number of players, played matches and observations for each playing position of four LIQUID-MOLY Handball Bundesliga teams across three seasons.

		All			With ball tracking		
Team	Position	Players	Matches	Observations	Players	Matches	Observations
A	Wing	6	95	288	6	76	227
	Center	13	95	640	13	76	506
	Pivot	6	95	220	6	76	176
B	Wing	5	89	276	5	69	212
	Center	22	89	575	22	69	453
	Pivot	3	89	213	3	69	161
C	Wing	12	96	326	11	41	142
	Center	24	96	606	24	41	246
	Pivot	6	96	223	6	41	100
D	Wing	6	98	311	6	47	146
	Center	13	98	651	13	47	330
	Pivot	5	98	245	5	47	110

Notes: One observation correspond to one player per match. Total number of matches (n=347) and players (n=109) differs to the column sum due to duplicated match IDs, when teams play against each other and player transfers.

### 2.3. Tracking system

A fixed installed LPS technology (Kinexon Perform LPS) was used, as described in detail elsewhere (21). The technology collected 2D positional and 3D IMU (accelerometer, gyroscope, and magnetometer) data at 20 and 100 Hz, respectively. Briefly, in all sports halls, 12 antennae were fix installed around the playing field connected to one base station. During the matches, the players wore a harness packed with a sensor at the center of the upper back, which transmitted time signals via radio technology to the antenna, and via a wide local area network to the base station. Additionally, a sensor was included in the balls to allow their tracking. The used technology has been considered as valid and reliable for movement patterns of indoor team sports including team handball (22–27). However, the metrics derived from a combination of LPS and IMU data (e.g., impacts and jumps) are less studied yet. Furthermore, the anchor and field placement could influence the LPS output (28).

### 2.4. Data analysis

Match summary statistic files (*n* = 394) were exported using the Kinexon system software (Kinexon Web Application, HBL-Cloud). The players were grouped into the following positions: wing (right and left winger), center (right and left back, center back), and pivot (16). The match outcomes were grouped into win, lost, and draw. After visiting the data, 11, 1, and 4 files were removed due to different column names, missing entries, and multiple entries of the same players, respectively. Thus, 378 files (347 matches) remained and were further checked for plausibility. In 134 matches, no ball was tracked, meaning that no passes and shots were reported. Thus, these matches were excluded from the throwing analysis. Additionally, two observations with 127 jumps and 65 impacts per match were removed due to implausible high counts. At least, all players with no playing time were excluded. Also, goalkeepers were excluded, because their clearly different technical-tactical demands compared to the other playing positions (12). To quantify the team performance, the sum of players for each physical variable was calculated, with exception of variables representing a maximum, where the mean of the players was used. Players that return after an injury were included in the data set. In total, 347 matches (213 with an additional ball tracking) with 109 different players were used for statistical analysis. A detailed description of all variables used in this study is shown in Table 1.

### 2.5. Statistical analysis

Data distribution were explored visually. To estimate differences between more than two groups (i.e., season, team, match outcome, and playing position), one-way ANOVAs on trimmed means (*tr* = 0.2) were calculated. The mean differences between halves were estimated using Yuens trimmed means test for paired samples (29). Effect sizes were computed, whereby $\;\;\hat{\;\;\xi }<0.30$, 0.30 to 0.50, and >0.50 correspond to small, medium, and large effects, respectively (29, 30). The results were reported with trimmed means and corresponding standard deviations as well as effect sizes from one-way ANOVA analysis. When large effects were detected by the ANOVA, pairwise comparisons were conducted on trimmed means. All mean differences $\;\;\hat{\;\;\psi }$ were reported with 95% confidence intervals. A multiple testing Bonferroni correction was applied. An alpha level of 0.05 was used for statistical significance. All calculations and visualizations were conducted in R version 4.2.0 (31–33).

## 3. Results

### 3.1. Effect of season

Table 3 shows the effects of the season on the physical match demands. There were medium and small effects ranging between $0.32\leq \;\;\hat{\;\;\xi }\leq 0.42$ and $0.09\leq \;\;\hat{\;\;\xi }\leq 0.24$, respectively. Large effects were found for the accumulated acceleration load ($\;\;\hat{\;\;\xi }=0.86$) and the maximum metabolic power ($\;\;\hat{\;\;\xi }=0.6$). The post-hoc tests show that the load was lower in 2019/2020 compared to 2020/2021 ($\;\;\hat{\;\;\psi }=-528.8\;[-575.1,-482.6]$) and 2021/2022 ($\;\;\hat{\;\;\psi }=-514.8\;[-565.7,-463.9]$). Also, the maximum metabolic power was lower in 2019/2020 ($\;\;\hat{\;\;\psi }=-6.06\;[-7.58,-4.53]$) and in 2020/2021 ($\;\;\hat{\;\;\psi }=-4.79\;[-6.09,-3.48]$) compared to 2021/2022.

**Table 3:** Physical match demands of four LIQUI-MOLY Handball Bundesliga teams across three seasons (*n*_all_ matches: 2019/2020 = 90, 2020/2021 = 136, 2021/2022 = 121; *n*_ball tracking_: 2019/2020 = 33, 2020/2021 = 61, 2021/2022 = 119). Values are expressed as trimmed means (trim = 0.2) and corresponding standard deviation of summarized team performance with exception of variables representing a maximum, where the mean was used.

Variable	Season 2019/2020	Season 2020/2021	Season 2021/2022	Effect size	95%CI effect size	Magnitude
Playing time [s]	21,191.5±270.3	21,377.2±240.2	21,401.9±248.0	0.42	[0.29, 0.54]	Medium
Distance [m]	28,331.6±1,045.5	28,680.9±1,104.0	28,665.1±1,115.0	0.18	[0.06, 0.33]	Small
Max. speed [km/h]	23.7±0.9	23.9±0.7	24.0±0.7	0.24	[0.13, 0.35]	Small
Time speed very high [s]	168.1±37.3	170.1±41.1	175.3±42.7	0.13	[0.04, 0.26]	Small
Time speed high [s]	920.7±98.9	921.2±115.0	921.4±102.9	0.10	[0.02, 0.23]	Small
Time speed medium [s]	1,861.3±190.7	1,914.3±152.9	1,909.6±169.6	0.19	[0.04, 0.31]	Small
Time speed low [s]	5,965.4±378.8	6,055.0±417.7	6,063.7±588.7	0.14	[0.03, 0.28]	Small
Time speed very low [s]	12,302.5±463.5	12,374.6±465.0	12,375.7±585.6	0.10	[0.02, 0.27]	Small
Max. acceleration [m/s${}^{2}$]	3.4±0.2	3.4±0.1	3.4±0.1	0.32	[0.14, 0.45]	Medium
Acceleration very high	31.4±12.0	30.9±11.5	33.3±10.4	0.13	[0.05, 0.26]	Small
Acceleration high	64.9±15.2	67.0±10.7	67.8±10.8	0.13	[0.05, 0.33]	Small
Acceleration medium	125.4±15.3	126.3±15.0	125.8±14.8	0.09	[0.01, 0.21]	Small
Acceleration low	168.4±22.0	173±17.3	167.2±18.6	0.20	[0.09, 0.31]	Small
Max. deceleration [m/s${}^{2}$]	−3.3±0.2	−3.3±0.2	−3.3±0.1	0.13	[0.03, 0.23]	Small
Deceleration very high	16.2±6.6	14.1±5.4	16.4±6.8	0.23	[0.08, 0.35]	Small
Deceleration high	37.5±10.0	37.3±9.5	39.2±9.7	0.14	[0.04, 0.29]	Small
Deceleration medium	90.4±17.0	92.7±14.1	91.0±11.5	0.12	[0.04, 0.24]	Small
Deceleration low	148.9±21.7	152.3±20.7	145.5±19.3	0.18	[0.05, 0.33]	Small
Max. metabolic power [W/kg]	61.9±4.7	63.2±4.2	67.9±4.4	0.60	[0.5, 0.69]	Large
Time metabolic power very high [s]	656.3±68.1	654.7±68.0	695.9±76.6	0.32	[0.15, 0.44]	Medium
Time metabolic power high [s]	1,070.0±126.0	1,095.1±118.7	1,094.8±104.4	0.14	[0.03, 0.26]	Small
Time metabolic power medium [s]	6,941.3±367.3	7,031.3±398.4	7,002.8±514.9	0.14	[0.04, 0.23]	Small
Time metabolic power low [s]	12,584±446.5	12,666.9±457.5	12,662.1±556.9	0.13	[0.04, 0.26]	Small
Accumulated acceleration load [a.u.]	2,964.3±140.1	3,493.2±125.4	3,479.1±146.8	0.86	[0.83, 0.89]	Large
Impacts	111.9±23.9	97.5±19.8	102.3±21.5	0.36	[0.23, 0.47]	Medium
Jumps	107.8±17.7	118.6±19.8	122.3±17.3	0.39	[0.29, 0.51]	Medium
Passes	669.5±86.8	689.1±85.8	667.4±124.3	0.21	[0.05, 0.44]	Small
Shots	50.1±7.0	51.2±8.8	51.2±8.4	0.17	[0.04, 0.38]	Small

Notes: CI, confidence interval.

### 3.2. Effect of team

**Figure 1:**
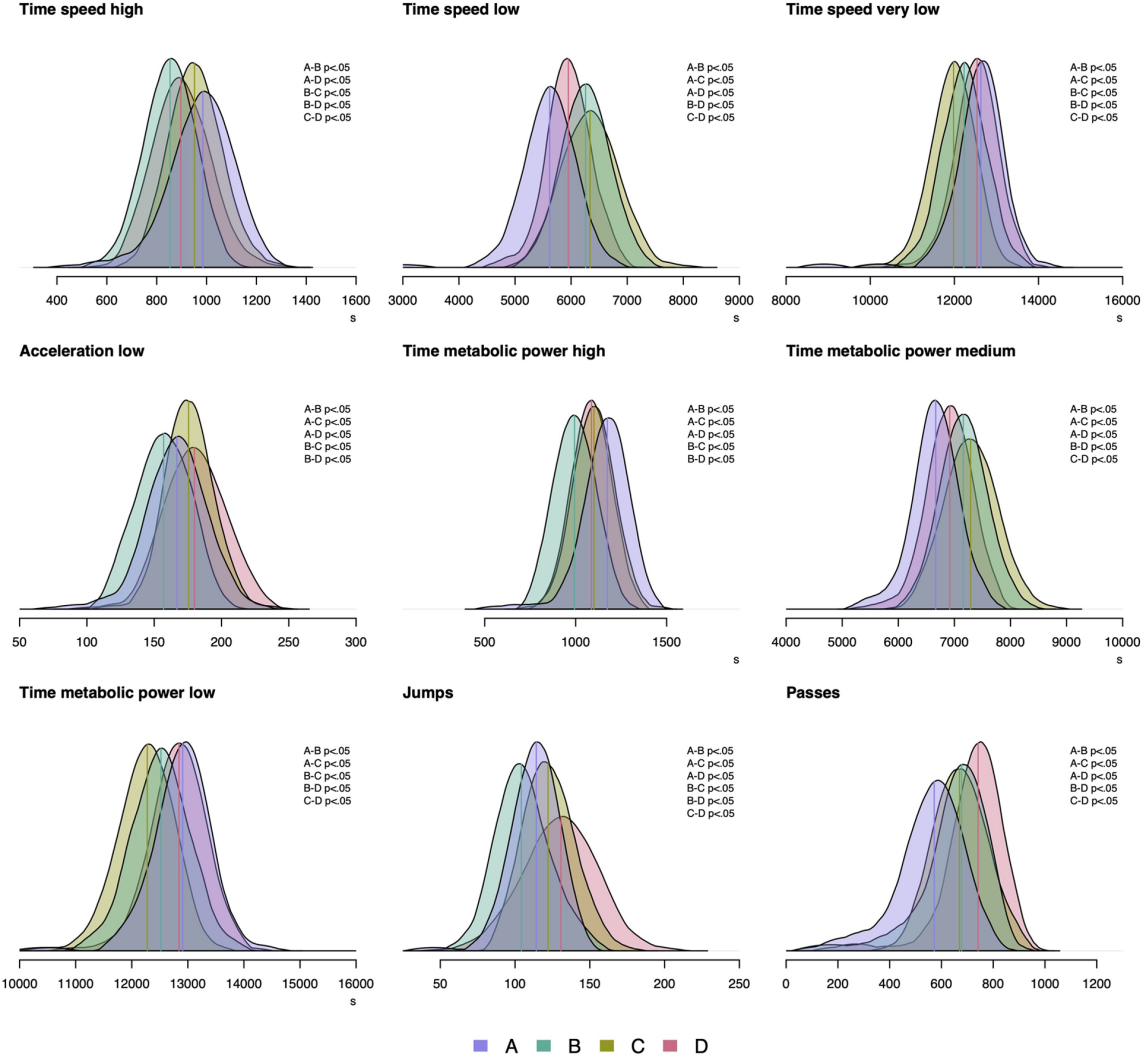
Selected distributions of physical match demands from the four LIQUI-MOLY Handball Bundesliga teams. Pairwise comparisons were done on trimmed means. Variables were selected by large effects resulting from ANOVA on trimmed means. Variables without unit are presented as count.

Table 4 shows the physical match demands of the four teams and the results from the ANOVA. There were large, medium, and small effects ranging between $0.56\leq \;\;\hat{\;\;\xi }\leq 0.72$, $0.30\leq \;\;\hat{\;\;\xi }\leq 0.46$, and $0.17\leq \;\;\hat{\;\;\xi }\leq 0.29$, respectively. Additionally, Figure 1 shows the distributions and results from the post-hoc tests of the variables with large effect sizes.

**Table 4:** Physical match demands of the four LIQUI-MOLY Handball Bundesliga teams. Values are expressed as trimmed means (trim = 0.2) and corresponding standard deviation of summarized team performance with exception of variables representing a maximum, where the mean was used.

Variable	A	B	C	D	Effect size	95%CI effect size	Magnitude
Playing time [s]	21,333.1±270.5	21,273±286.4	21,308.9±254.0	21,442.5±246.6	0.28	[0.17, 0.43]	Small
Distance [m]	28,681.4±956.4	28,042.2±1,062.8	29,169.9±1,085.1	28,472.6±1,010.0	0.46	[0.33, 0.54]	Medium
Max. speed [km/h]	23.7±0.9	24.0±0.9	24.0±0.7	23.9±0.7	0.20	[0.07, 0.34]	Small
Time speed very high [s]	184.9±42.3	166.7±41.1	178.9±31.6	154.0±37.8	0.34	[0.23, 0.46]	Medium
Time speed high [s]	986.4±101.9	853.9±86.0	951.6±84.6	896.4±94.6	0.56	[0.45, 0.64]	Large
Time speed medium [s]	1,981.8±157.1	1,799±156.4	1,893.9±142.6	1,928.9±153.1	0.46	[0.38, 0.57]	Medium
Time speed low [s]	5,621.8±348.5	6,260.7±369.7	6,339.3±428.7	5,949.3±321.5	0.72	[0.64, 0.80]	Large
Time speed very low [s]	12,639.4±422.4	12,234.2±431.5	11,981.9±412.9	12,534.7±420.2	0.60	[0.52, 0.67]	Large
Max. acceleration [m/s${}^{2}$]	3.4±0.1	3.4±0.2	3.4±0.1	3.4±0.1	0.17	[0.06, 0.28]	Small
Acceleration very high	29.4±11.1	33.5±12.3	35.6±9.9	29.4±10.8	0.28	[0.16, 0.41]	Small
Acceleration high	63.9±10.4	67.5±10.7	70.9±10.8	65.2±12.5	0.29	[0.18, 0.42]	Small
Acceleration medium	123.6±14.7	121.6±15.0	127.4±11.7	131.1±15.6	0.30	[0.17, 0.42]	Medium
Acceleration low	166.8±17.1	157.1±18.5	175.6±15.0	179.7±19.7	0.56	[0.45, 0.67]	Large
Max. deceleration [m/s${}^{2}$]	−3.2±0.1	−3.3±0.2	−3.3±0.1	−3.3±0.2	0.32	[0.18, 0.45]	Medium
Deceleration very high	14.9±5.6	14.4±6.6	16.4±6.9	16.2±6.3	0.18	[0.08, 0.29]	Small
Deceleration high	37.5±9.6	35.9±9.9	40.3±10.2	38.5±8.6	0.20	[0.11, 0.33]	Small
Deceleration medium	90.9±12.0	90.5±14.8	96.5±15.2	89.0±12.8	0.28	[0.14, 0.40]	Small
Deceleration low	152.4±18.2	141.8±16.4	161.2±18.2	141.9±18.7	0.46	[0.38, 0.54]	Medium
Max. metabolic power [W/kg]	63.6±4.8	63.3±5.7	66.0±4.5	65.1±5.2	0.25	[0.15, 0.37]	Small
Time metabolic power very high [s]	691.3±72.1	636.8±65.0	698.3±65.0	648.7±63.7	0.42	[0.31, 0.52]	Medium
Time metabolic power high [s]	1,173.7±98.3	993.9±101.9	1,100.6±90.1	1,087.3±89.0	0.65	[0.55, 0.73]	Large
Time metabolic power medium [s]	6,665.1±331.0	7,160.2±353.3	7,291.2±407.3	6,917.4±338.3	0.63	[0.57, 0.72]	Large
Time metabolic power low [s]	12,904.4±406.4	12,518.8±417.7	12,280.1±393.0	12,843.3±402.8	0.60	[0.53, 0.68]	Large
Accumulated acceleration load [a.u.]	3,421.8±222.4	3,333.3±260.0	3,323.7±229.7	3,520.9±241.8	0.33	[0.21, 0.44]	Medium
Impacts	112.9±19.8	92.9±19.3	102.9±23.9	101.9±19.5	0.39	[0.30, 0.48]	Medium
Jumps	114.1±13.3	104.1±15.4	122.1±16.0	130.8±20.1	0.60	[0.52, 0.70]	Large
Passes	572.2±86.2	677.5±74.2	669.4±85.3	740.8±75.6	0.68	[0.48, 0.84]	Large
Shots	53.8±8.5	50.8±7.7	51.7±6.4	48.9±7.7	0.29	[0.12, 0.49]	Small

Notes: CI, confidence interval.

The mean differences of post-hoc test associated with Table 4 show that compared to Team B, Team A showed higher values for time at high ($\;\;\hat{\;\;\psi }=132.5\;[93.4,171.5]$) and very-low speed ($\;\;\hat{\;\;\psi }=405.2\;[233.6,576.8]$), and numbers of low acceleration ($\;\;\hat{\;\;\psi }=9.6\;[2.2,17.1]$), time at high ($\;\;\hat{\;\;\psi }=179.8\;[139.5,220.1]$) and low metabolic power ($\;\;\hat{\;\;\psi }=385.6\;[223.3,547.9]$), and jumps ($\;\;\hat{\;\;\psi }=10.0\;[4.4,15.6]$). Lower values were found for them for time at low speed ($\;\;\hat{\;\;\psi }=-638.9\;[-780.2,-497.6]$), time at medium metabolic power ($\;\;\hat{\;\;\psi }=-495.1\;[-623.1,-367.2]$), and passes ($\;\;\hat{\;\;\psi }=-105.3\;[-157.2,-53.4]$).

Compared to Team C, Team A had higher values for time at very-low speed ($\;\;\hat{\;\;\psi }=657.5\;[489.3,825.7]$), time at high ($\;\;\hat{\;\;\psi }=73.1\;[34.3,111.9]$) and low metabolic power ($\;\;\hat{\;\;\psi }=624.3\;[464.4,784.1]$). Lower values were found for them for time at low speed ($\;\;\hat{\;\;\psi }=-717.6\;[-880.6,-554.6]$), numbers of low acceleration ($\;\;\hat{\;\;\psi }=-8.8\;[-15.4,-2.3]$), time at medium metabolic power ($\;\;\hat{\;\;\psi }=-626.1\;[-776.7,-475.5]$), jumps ($\;\;\hat{\;\;\psi }=-8.0\;[-13.9,-2.0]$), and passes ($\;\;\hat{\;\;\psi }=-97.2$ [−146.7,−47.7]).

Compared to Team D, Team A had higher values for time at high speed ($\hat{\psi }=89.9\;[48.9,130.0]$) and time at high metabolic power ($\;\;\hat{\;\;\psi }=86.4\;[48.9,123.9]$). Lower values were found for them for time at low speed ($\;\;\hat{\;\;\psi }=-327.5\;[-462.4,-192.7]$), numbers of low acceleration ($\;\;\hat{\;\;\psi }=-12.9\;[-20.5,-5.3]$), time at medium metabolic power ($\;\;\hat{\;\;\psi }=-252.3\;[-383.4,-121.3]$), jumps ($\;\;\hat{\;\;\psi }=-16.7\;[-23.6,-9.8]$), and passes ($\;\;\hat{\;\;\psi }=-168.6$ [−213.9,−123.3]).

Compared to Team C, Team B had higher values for time at very-low speed ($\;\;\hat{\;\;\psi }=252.3\;[73.5,431.0]$) and time at low metabolic power ($\;\;\hat{\;\;\psi }=238.6\;[66.3,410.9]$). Lower values were found for them for time at high speed ($\;\;\hat{\;\;\psi }=-97.7$ [−133.9,−61.5]), numbers of low acceleration ($\;\;\hat{\;\;\psi }=-18.5$ [−25.4,−11.6]), time at high metabolic power zone ($\;\;\hat{\;\;\psi }=-106.7\;[-146.1,-67.2]$), and jumps ($\;\;\hat{\;\;\psi }=-17.9\;[-24.2,-11.7]$).

Compared to Team D, Team B had higher values for time at low speed ($\;\;\hat{\;\;\psi }=311.4\;[173.1,449.6]$) and time at medium metabolic power ($\;\;\hat{\;\;\psi }=242.8\;[108.1,377.5]$). Lower values were found for them for time at high ($\;\;\hat{\;\;\psi }=-42.5\;[-80.6,-4.5]$) and very-low speed ($\;\;\hat{\;\;\psi }=-300.5\;[-471.1,-129.9])$. Also, lower values were found in number of low acceleration ($\;\;\hat{\;\;\psi }=-22.6$ [−30.5,−14.7]), time at high ($\;\;\hat{\;\;\psi }=-93.3\;[-131.5,-55.2]$) and low metabolic power ($\;\;\hat{\;\;\psi }=-324.5\;[-490.5,-158.5]$), jumps ($\;\;\hat{\;\;\psi }=-26.7\;[-33.8,-19.5]$), and passes ($\;\;\hat{\;\;\psi }=-63.3$ [−106.1,−20.4]).

Compared to Team D, Team C had higher values for time at high ($\;\;\hat{\;\;\psi }=55.1\;[16.8,93.5]$) and low speed ($\;\;\hat{\;\;\psi }=390.0$ [229.6,550.5]) and time at medium metabolic power ($\;\;\hat{\;\;\psi }=373.8$ [217.5,530.0]). Lower values were found for them for time at very-low speed ($\;\;\hat{\;\;\psi }=-552.9\;[-719.9,-385.7]$), time at low metabolic power ($\;\;\hat{\;\;\psi }=-563.1\;[-726.8,-399.5]$), jumps ($\;\;\hat{\;\;\psi }=-8.7$ [−16.1,−1.3]), and passes ($\;\;\hat{\;\;\psi }=-71.4$ [−110.8,−31.9]).

### 3.3. Effect of match outcome

Table 5 summarizes the effects of the match outcome on the physical match demands. There were medium and small effects ranging between $0.31\leq \;\;\hat{\;\;\xi }\leq 0.36$ and $0.17\leq \;\;\hat{\;\;\xi }\leq 0.27$, respectively.

**Table 5:** Physical match demands of four LIQUI-MOLY Handball Bundesliga teams dependent on the match outcome (*n*_all_ matches: win = 196, draw = 28, lost = 147; *n*_ball tracking_: win = 125, draw = 17, lost = 86). Values are expressed as trimmed means (trim = 0.2) and corresponding standard deviation of summarized team performance with exception of variables representing a maximum, where the mean was used.

Variable	Win	Draw	Lost	Effect size	95%CI effect size	Magnitude
Playing time [s]	21,324.1±266.6	21,287.9±277.7	21,371.9±267.8	0.19	[0.06, 0.44]	Small
Distance [m]	28,572.2±1085.8	28,355.7±1364.3	28,626.0±1061.7	0.20	[0.03, 0.44]	Small
Max. speed [km/h]	23.9±0.7	23.5±1	23.9±0.8	0.31	[0.08, 0.57]	Medium
Time speed very high [s]	177.1±40.7	155.7±40.1	166.4±37.9	0.27	[0.08, 0.52]	Small
Time speed high [s]	926.7±106.0	897.6±99.9	916.0±106.7	0.20	[0.03, 0.44]	Small
Time speed medium [s]	1,872.9±164.6	1,848.5±183.6	1,941.9±154.4	0.33	[0.08, 0.55]	Medium
Time speed low [s]	6,038.3±538.1	6,141.9±389.2	6,015.7±390.8	0.22	[0.03, 0.46]	Small
Time speed very low [s]	12,352.4±558.4	12,286.0±546.0	12,363.5±449.2	0.19	[0.03, 0.39]	Small
Max. acceleration [m/s${}^{2}$]	3.4±0.1	3.4±0.2	3.4±0.1	0.24	[0.06, 0.53]	Small
Acceleration very high	33.0±10.8	28.9±12.3	30.8±10.6	0.23	[0.07, 0.48]	Small
Acceleration high	67.5±11.4	65.6±8.5	66.1±11.2	0.17	[0.03, 0.41]	Small
Acceleration medium	125.1±13.6	126.6±16.5	127.0±15.6	0.18	[0.04, 0.38]	Small
Acceleration low	166.1±18.1	167.0±16.3	175.9±19.4	0.36	[0.13, 0.66]	Medium
Max. deceleration [m/s${}^{2}$]	−3.3±0.1	−3.3±0.2	−3.3±0.1	0.19	[0.03, 0.40]	Small
Deceleration very high	16.0±6.5	14.7±6.8	14.9±6.6	0.19	[0.04, 0.47]	Small
Deceleration high	38.9±8.8	36.5±11.2	37.1±10.0	0.19	[0.05, 0.44]	Small
Deceleration medium	90.9±12.3	90.3±13.3	92.6±15.6	0.20	[0.04, 0.41]	Small
Deceleration low	147.1±18.7	145.1±18.5	152.4±22.9	0.21	[0.04, 0.42]	Small
Max. metabolic power [W/kg]	64.6±4.5	63.2±6.1	64.4±6.1	0.24	[0.05, 0.56]	Small
Time metabolic power very high [s]	674.8±70.2	647.6±70.4	663.5±77.9	0.26	[0.05, 0.45]	Small
Time metabolic power high [s]	1,079.7±120.0	1,058.7±116.4	1,104.4±105.8	0.27	[0.05, 0.48]	Small
Time metabolic power medium [s]	7,002.2±484.0	7,081.0±475.0	6,990.0±362.4	0.21	[0.03, 0.40]	Small
Time metabolic power low [s]	12,631.9±536.1	12,591.3±555.5	12,657.5±436.3	0.19	[0.04, 0.41]	Small
Accumulated acceleration load [a.u.]	3,392.5±236.3	3,345.7±270.6	3,409.7±274.3	0.18	[0.04, 0.43]	Small
Impacts	104.7±21.8	105.1±17.1	99.2±21.9	0.22	[0.06, 0.45]	Small
Jumps	118.9±18.2	114.1±22.6	116.0±19.4	0.19	[0.06, 0.43]	Small
Passes	659.9±103.1	711.1±68.9	688.3±113.1	0.34	[0.04, 0.57]	Medium
Shots	52.2±8.2	50.2±5.3	49.4±8.1	0.25	[0.03, 0.63]	Small

Notes: CI, confidence interval.

### 3.4. Effect of playing position

**Figure 2:**
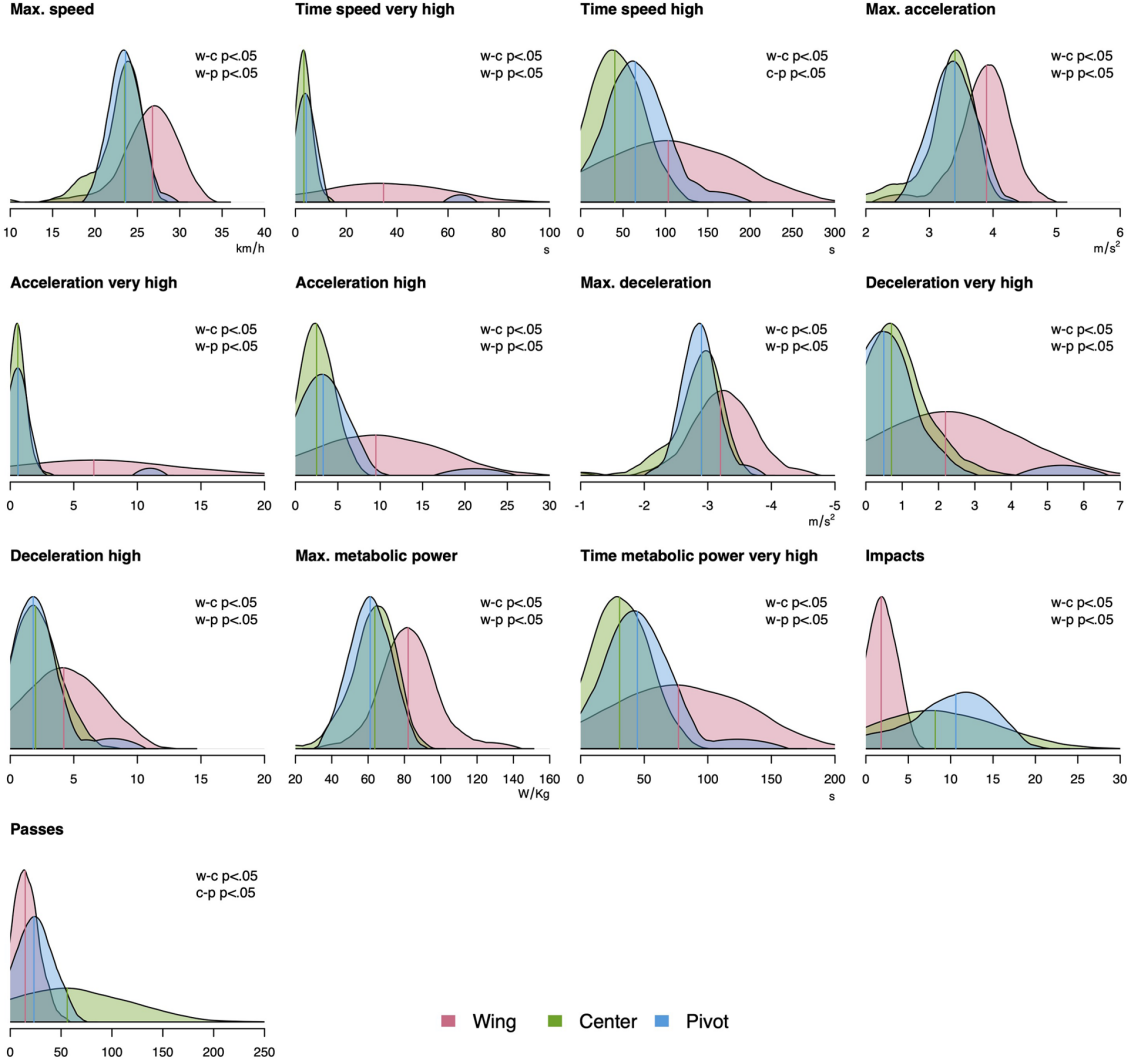
Selected distributions of physical match demands from handball player of four LIQUI-MOLY Handball Bundesliga teams depending on the playing position. Pairwise comparisons were done on trimmed means. Variables were selected by large effects resulting from ANOVA on trimmed means. Variables without unit are presented as count.

Table 6 shows the effects of the playing position on the physical match demands and the corresponding results from the ANOVA. There were large, medium, and small effects ranging between $0.64\leq \;\;\hat{\;\;\xi }\leq 0.98$, $0.30\leq \hat{\xi }\leq 0.48$, and $0.22\leq \;\;\hat{\;\;\xi }\leq 0.29$, respectively. Figure 2 displays differences in the distribution of the variables and summarizes the results from post-hoc tests for all large effect sizes.

**Table 6:** Physical match demands of four LIQUI-MOLY Handball Bundesliga teams dependent on the playing position. Values are expressed as trimmed means (trim = 0.2) and corresponding standard deviation.

Variable	Winger	Center	Pivot	Effect size	95%CI effect size	Magnitude
Playing time [s]	1,784.5±1086.2	1,158.7±872.0	1,721.4±619.5	0.38	[0.16, 0.67]	Medium
Distance [m]	2,384.6±1,389.9	1,610.4±1,162.0	2,182.7±782.0	0.34	[0.09, 0.65]	Medium
Max. speed [km/h]	26.8±2.1	23.5±1.6	23.6±1.2	0.82	[0.59, 1.01]	Large
Time speed very high [s]	34.7±19.7	3.3±2.4	4.2±2.9	0.86	[0.69, 1.44]	Large
Time speed high [s]	103.2±67.3	40.4±27.3	64.5±26.9	0.72	[0.37, 1.01]	Large
Time speed medium [s]	132.8±79.6	116.4±86.4	137.6±53.4	0.22	[0.07, 0.46]	Small
Time speed low [s]	398.4±247.2	377.2±281.5	471.8±175.8	0.26	[0.06, 0.55]	Small
Time speed very low [s]	1,101.0±695.1	625.7±492.7	1,029.8±393.6	0.46	[0.21, 0.65]	Medium
Max. acceleration [m/s${}^{2}$]	3.9±0.3	3.4±0.2	3.4±0.3	0.80	[0.55, 1.00]	Large
Acceleration very high	6.6±4.7	0.6±0.5	0.6±0.6	0.98	[0.71, 1.51]	Large
Acceleration high	9.5±5.9	2.5±1.6	3.3±2.1	0.80	[0.51, 1.15]	Large
Acceleration medium	11.8±7.2	6.4±4.2	9.2±3.9	0.48	[0.21, 0.78]	Medium
Acceleration low	11.9±8.4	10.3±7.2	13.3±5.4	0.27	[0.04, 0.53]	Small
Max. deceleration [m/s${}^{2}$]	−3.2±0.3	−2.9±0.3	−2.9±0.2	0.64	[0.26, 0.89]	Large
Deceleration very high	2.2±1.5	0.7±0.6	0.5±0.5	0.77	[0.44, 1.01]	Large
Deceleration high	4.2±2.5	2.0±1.5	1.8±1.1	0.64	[0.24, 0.89]	Large
Deceleration medium	7.7±4.4	5.3±3.5	5.8±2.7	0.38	[0.09, 0.74]	Medium
Deceleration low	11.3±7.0	8.7±6.3	11.3±4.9	0.30	[0.06, 0.52]	Medium
Max. metabolic power [W/kg]	82.0±10.9	63.8±9.7	61.2±8.9	0.83	[0.65, 1.01]	Large
Time metabolic power very high [s]	76.8±44.7	30.6±19.5	44.7±17.7	0.70	[0.42, 1.00]	Large
Time metabolic power high [s]	88.0±54.7	61.6±46.1	79.6±32.2	0.32	[0.07, 0.62]	Medium
Time metabolic power medium [s]	488.5±293.0	429.1±318.1	538.7±193.3	0.28	[0.08, 0.62]	Small
Time metabolic power low [s]	1,120.8±705.7	644.0±504.8	1,053.7±400.3	0.44	[0.14, 0.74]	Medium
Accumulated acceleration load [a.u.]	251.4±143.7	194.0±135.6	277.0±107.7	0.30	[0.11, 0.54]	Medium
Impacts	1.8±1.5	8.2±6.3	10.6±3.4	0.67	[0.55, 0.75]	Large
Jumps	4.6±3.5	8.6±6.5	6.5±3.6	0.46	[0.14, 0.76]	Medium
Passes	14.8±10.8	55.9±51.4	23.3±15.1	0.89	[0.41, 1.18]	Large
Shots	2.8±2.1	3.1±2.9	2.4±1.2	0.29	[0.06, 0.50]	Small

Notes: CI, confidence interval.

The mean differences of post-hoc test associated with Figure 2 show that compared to centers, wingers showed higher values for maximum running speed ($\;\;\hat{\;\;\psi }=3.3\;[2.0,4.5]$), time at very-high ($\;\;\hat{\;\;\psi }=31.4\;[21.3,41.5]$) and high speed ($\;\;\hat{\;\;\psi }=62.7\;[22.8,102.7]$), maximum acceleration ($\;\;\hat{\;\;\psi }=0.5\;[0.4,0.7]$), numbers of very-high ($\;\;\hat{\;\;\psi }=6.1\;[3.5,8.7]$) and high acceleration ($\;\;\hat{\;\;\psi }=7.1\;[3.7,10.4$]), maximum deceleration ($\;\;\hat{\;\;\psi }=-0.3\;[-0.5,-0.1]$), and numbers of very-high ($\;\;\hat{\;\;\psi }=1.5\;[0.7,2.3]$) and high deceleration ($\;\;\hat{\;\;\psi }=2.3\;[0.8,3.7]$). Additionally, wingers had higher values for maximum ($\;\;\hat{\;\;\psi }=18.2\;[12.1,24.3]$) and time at very-high metabolic power ($\;\;\hat{\;\;\psi }=46.2\;[20.0,72.4$]. Also, lower values were found for them for numbers of impacts ($\;\;\hat{\;\;\psi }=-6.3$ [−8.5,−4.2]) and passes $\;\;\hat{\;\;\psi }=-41.1\;[-60.4,-21.7]$).

Compared to pivots, wingers had higher values for maximum running speed ($\;\;\hat{\;\;\psi }=3.3\;[1.9,4.6]$), time at very-high ($\;\;\hat{\;\;\psi }=30.5$ [20.3,40.7]) and maximum acceleration ($\;\;\hat{\;\;\psi }=0.52\;[0.3,0.7]$), numbers of very-high ($\;\;\hat{\;\;\psi }=6.0\;[3.4,8.6]$) and high acceleration ($\;\;\hat{\;\;\psi }=6.3\;[2.8,9.8]$), maximum deceleration ($\;\;\hat{\;\;\psi }=-0.37$ [−0.6,−0.2]), and numbers of very-high ($\;\;\hat{\;\;\psi }=1.7\;[0.9,2.6]$) and high deceleration ($\;\;\hat{\;\;\psi }=2.4\;[0.9,3.9]$). Also, wingers had higher values for maximum metabolic power ($\;\;\hat{\;\;\psi }=20.8$ [12.8,28.9]) and time at very-high metabolic power ($\;\;\hat{\;\;\psi }=32.1\;[4.3,59.9]$). Furthermore, lower values were detected for them regarding numbers of impacts ($\;\;\hat{\;\;\psi }=-8.7$ [−12.3,−5.1]).

Related to pivots, centers had higher values for passes $\;\;\hat{\;\;\psi }=32.6\;[11.8,53.4]$) and lower for time at high speed ($\;\;\hat{\;\;\psi }=-24.1\;[-41.3,-6.9]$).

### 3.5. Effect of halftime

Table 7 shows the effects of the halftime on the physical match demands. Medium and small effects were observed ranging between $0.37\leq \;\;\hat{\;\;\xi }\leq 0.47$ and $0.00\leq \;\;\hat{\;\;\xi }\leq 0.27$, respectively.

**Table 7:** Physical match demands of four LIQUI-MOLY Handball Bundesliga teams dependent on the halftime. Values are expressed as trimmed means (trim = 0.2) and corresponding standard deviation of summarized team performance with exception of variables representing a maximum, where the mean was used.

Variable	1.HT	2.HT	95%CI mean diff	Effect size	Magnitude
Playing time [s]	10,679.2±147.0	10,678.7±160.5	[−19.95, 20.92]	0.00	Small
Distance [m]	14,315.1±684.5	14,281.8±649.6	[−50.98, 117.65]	0.04	Small
Max. speed [km/h]	24.1±0.9	23.9±0.9	[0.09, 0.35]	0.17	Small
Time speed very high [s]	92.1±23.8	78.6±23.5	[10.66, 16.38]	0.38	Medium
Time speed high [s]	482.8±66.0	435.7±63.9	[38.87, 55.37]	0.47	Medium
Time speed medium [s]	945.2±95.4	952.9±112.2	[−20.91, 5.49]	0.05	Small
Time speed low [s]	2,947.4±249.9	3,093.1±287.4	[−176.32, −115.14]	0.37	Medium
Time speed very low [s]	6,237.9±302.7	6,128.3±322.1	[69.22, 149.85]	0.25	Small
Max. acceleration [m/s${}^{2}$]	3.4±0.2	3.4±0.2	[0.01, 0.05]	0.12	Small
Acceleration very high	16.7±6.8	15.0±6.5	[0.97, 2.43]	0.20	Small
Acceleration high	34.5±7.4	32.0±7.7	[1.51, 3.49]	0.23	Small
Acceleration medium	64.4±9.7	61.1±9.6	[1.93, 4.70]	0.24	Small
Acceleration low	86.0±12.4	84.1±12.7	[0.24, 3.51]	0.11	Small
Max. deceleration [m/s${}^{2}$]	−3.3±0.2	−3.3±0.2	[−0.08, −0.02]	0.19	Small
Deceleration very high	7.8±4.6	7.3±3.8	[−0.13, 1.13]	0.08	Small
Deceleration high	19.9±6.0	17.7±6.0	[1.44, 3.07]	0.27	Small
Deceleration medium	47.0±9.0	44.1±9.3	[1.63, 4.22]	0.23	Small
Deceleration low	77.0±13.2	72.0±11.8	[3.38, 6.79]	0.27	Small
Max. metabolic power [W/kg]	64.9±5.7	64.3±6.1	[−0.14, 1.28]	0.07	Small
Time metabolic power very high [s]	349.2±48.4	318.4±40.1	[25.25, 36.31]	0.47	Medium
Time metabolic power high [s]	553.2±67.9	535.9±70.0	[8.95, 25.63]	0.17	Small
Time metabolic power medium [s]	3,433.0±229.6	3,568.1±266.4	[−163.64, −106.62]	0.38	Medium
Time metabolic power low [s]	6,375.6±290.2	6,277.6±316.8	[58.85, 137.14]	0.23	Small
Accumulated acceleration load [a.u.]	1,691.9±138.7	1,697.1±136.8	[−15.5, 5.16]	0.02	Small
Impacts	52.7±12.1	50.1±12.7	[1.28, 3.93]	0.15	Small
Jumps	57.9±10.9	59.6±10.6	[−2.81, −0.43]	0.11	Small
Passes	332.3±55.5	343.8±61.4	[−20.97, −1.98]	0.14	Small
Shots	25.4±4.4	26.0±4.6	[−1.33, 0.16]	0.09	Small

CI, confidence interval.

## 4. Discussion

The aim of this study was to investigate the physical match demands of four LIQUI-MOLY Handball-Bundesliga teams across three seasons with respect to the effects of season, team, match outcome, playing position, and halftime. Our main findings show that there were up to large effects concerning the (i) season, (ii) team, and (iii) playing position. Up to medium effects were evident concerning the (iv) match outcome and (v) halftime.

The first main finding was that there were up to large effects concerning the season (Table 3). In fact, there was a large increase in the accumulated acceleration load from the season 2019/2020 to both consecutive seasons and an increase in maximum metabolic power in season 2021/2022. Our findings support the assumption that the match demands have increased in top-level team handball (2, 4, 5) and provide, for the first time, quantitative evidence for such a progress. Due to the calculation of the accumulated acceleration load as an overall acceleration-based metric (Table 1), its large effect may be caused by the medium differences observed in the playing time and time spent at very-high metabolic power as well as number of impacts and jumps (Table 3). In that context, one explanation for the lower accumulated acceleration load in 2019/2020 is that, during Covid-19 pandemic, players have dropped out and player rotation strategies may have changed, which could both influence the team performance. A further interesting observation was that the reduced number of matches played in the season 2019/2020 (Table 3) caused by the pandemic had no negative physical impact on the following seasons. Comparable results are reported concerning instructed home-based strength and endurance program in team handball players (34, 35). Taken overall, the results suggest that top-level team handball has changed into a more dynamic team sport. However, more longitudinal studies are needed to clarify our observations.

In team handball, the success, expressed by the match outcome, is determined by multiple factors (1). In this context, we discuss the effects of team and match outcome together. We found up to large and medium effects concerning the team (Figure 1 and Table 1) and match outcome (Table 5), respectively. While previous studies reported no differences in physical match demands between top- and lower-ranked or winning and losing teams (12, 13), another study showed that on the European Championship 2020 and World Championships Qatar 2015 the winning teams conducted more metabolic power associated running activities compared to losing teams (15). With respect to high-intensity activities, our outcomes support those of (15), namely that the higher-ranked team (Team A) spent more time at high-speed and high metabolic power, whereas they executed a smaller number of jumps and passes (Figure 1 and Table 4) compared to the lower ranked teams (Team B, C, and D). In this context, winning teams perform less passes than drawing teams, but do not differ in terms of high-intensity running actions (Table 5). These findings may provide a new perspective of the influence of physical match demands on success in top-level team handball. It can be hypothesized that all the players are physically well developed (12) and technical-tactical actions are more important to win a match at this top-level. In conclusion, our data suggest that the running related physical match demands alone are not suitable to explain the match outcomes in top-level team handball. Thus, more studies that consider the technical-tactical context to explain the success are needed.

The third main finding was that small to large effects of playing position on physical match demands were present (Figure 2 and Table 6). Concretely, wingers showed the highest amount of high-intensity runs, pivots received the most impacts, and center throwed the most passes (Figure 2 and Table 6). These findings are consistent with those from previous studies (9, 10, 16, 36), showing that pivots cover the shortest distance at sprinting, but still operate at high-intensity due to high number of impacts (10, 36). In contrast, wingers show the lowest number of impacts (10) and perform together with center backs the greatest amount of high-intensity runs (12, 16, 35, 36). Additionally, backs are characterized by a substantially higher number of passes and shots (10). One well-accepted explanation for the differences is related to the different technical-tactical roles of the playing positions (10). Wingers cover longer distances back in the defense compared to center and pivots, and are the favored pass recipient in fast break situations in offensive situations. Our results underline the high-speed running and acceleration capacities of wingers and the high passing demands of center players (Table 6). Future studies should examine how these results could be successfully transferred into position-specific training. In summary, existing position differences in physical match demands indicate that there are highly specific position roles in top-level team handball.

The last main finding was that there are up to medium effects of the halftime on physical match demands (Table 7). In detail, time spent at very-high speed and very-high metabolic power decreased from first to second half. These findings are in line with those from previous studies, which investigate differences between halftimes during matches in men’s Portuguese and Danish top-level handball leagues (3, 17, 18). These previous studies show that physical match demands, which are related to high-intensity movements such as fast-running (17) or one-on-one situations (3), are decreasing from the first to the second half. Also, studies show that fatigue may occurs in certain phases of a match, mainly towards the end of the halftimes (2, 18). Furthermore, recent studies show position-specific fatigue effects (16, 37). Explanations for lower high intensity running activities in the second half in our study may be related to the scoreline and subsequent tactical changes (e.g., formation and player rotation) or individual fatigue. However, it is noteworthy that fatigue can be caused by numerous central and peripheral factors (38), which remained unknown in observational studies as conducted here. For the clarification, if differences in high-intensity actions between halftimes are caused by fatigue, additional standardized maximal muscular tests combined with electrophysiological measures are needed (38, 39). In summary, high-intensity running activities are lower in the second compared to the first half. The underlining technical-tactical or physiological causes remained unknown and need further studies.

## 5. Limitations

There are a few limitations, which should be mentioned. Normalizing the data to playing time concerning position differences might lead to different results (12). However, we decided to stay close to the raw values, because we could not consider offense and defense match segments. Also, we decided to use an ordinal scale for the match outcome. Reducing success to an ordinal scale may have led to a loss of information. Our decision based on the fact that a basic descriptive analysis, as conducted here, is the first step before building more complex models. Furthermore, studies indicate that the metabolic power model by Osgnach et al. (19) could underestimate the true energy expenditure (40, 41), which could lead to misinterpretations. At least deep insights into the calculation methods of certain variables (e.g., jumps, shots, passes, and impacts) could not be reported due to property rights. Therefore, more studies are needed that investigate the validity of the metabolic power approach (42) and the detected throws, impacts, jumps (43), and change of directions grounding on tracking based measures. All points can be considered by more detailed studies in the future.

## 6. Conclusion

For the first time, we provide a comprehensive analysis of physical match demands in handball players competing in the LIQUI-MOLY Handball-Bundesliga. We found that physical match demands differ on that top-level with up to large effect sizes concerning the season, team, match outcome, playing position, and halftime. Our outcomes can help practitioners and researchers to develop team and player profiles as well as to optimize talent identification, training, regeneration, prevention, and rehabilitation procedures, and gives numerous indications for future research directions.

## Data Availability

The data analyzed in this study is subject to the following licenses/restrictions: The collected data is freely available for each team competing in the LIQUI-MOLY Handball Bundesliga. Requests to access these datasets should be directed to info@liquimoly-hbl.de.

## References

[1] Wagner H Finkenzeller T Würth S von Duvillard SP. Individual, team performance in team-handball: a review. J Sports Sci Med. (2014) 13:808–16. PMID: ; PMCID: 25435773 PMC4234950

[2] Michalsik LB Madsen K Aagaard P. Physiological capacity, physical testing in male elite team handball. J Sports Med Phys Fitness. (2015) 55:415–29. PMID: 24402441

[3] Póvoas SC Seabra AF Ascensão AA Magalhães J Soares JM Rebelo AN. Physical, physiological demands of elite team handball. J Strength Cond Res. (2012) 26(12):3365–75. 10.1519/JSC.0b013e318248aeee 22222325

[4] Bilge M. Game analysis of olympic, world and european championships in men’s handball. J Hum Kinet. (2012) 35:109–18. 10.2478/v10078-012-0084-7 23486176 PMC3588687

[5] Meletakos GP Noutsos SK Bayios AI. Stable and changing characteristics of high-level handball as evidenced from world men’s championships. J Phys Educ Sport. (2020) 20:1354–61. 10.7752/jpes.2020.03187

[6] Pino-Ortega J Oliva-Lozano J Gantois P Nakamura FY Rico-González M. Comparison of the validity and reliability of local positioning systems against other tracking technologies in team sport: A systematic review. Proc Inst Mech Eng P J Sport Eng Technol. (2021) 236:73–82. 10.1177/175433712098823

[7] Torres-Ronda L Beanland E Whitehead S Sweeting A Clubb J. Tracking systems in team sports: a narrative review of applications of the data and sport specific analysis. Sports Med Open. (2022) 8:15. 10.1186/s40798-022-00408-z 35076796 PMC8789973

[8] van den Tillaar R Bhandurge S Stewart T. Can machine learning with imus be used to detect different throws and estimate ball velocity in team handball? Sensors. (2021) 21:7. 10.3390/s21072288 PMC8036950 33805871

[9] Font R Karcher C Reche X Carmona G Tremps V Irurtia A. Monitoring external load in elite male handball players depending on playing positions. Biol Sport. (2021) 38:475–81. 10.5114/biolsport.2021.101123 34475629 PMC8329973

[10] Karcher C Buchheit M. On-court demands of elite handball, with special reference to playing positions. Sports Med. (2014) 44:797–814. 10.1007/s40279-014-0164-z 24682948

[11] García-Sánchez C Navarro RM Karcher C. Physical demands during official competitions in elite handball: a systematic review. Int J Environ Res Public Health. (2023) 20:30. 10.3390/ijerph20043353 PMC9965087 36834047

[12] Manchado C Pueo B Chirosa-Rios LJ Tortosa-Martínez J. Time-motion analysis by playing positions of male handball players during the european championship 2020. Int J Environ Res Public Health. (2021) 18:15. 10.3390/ijerph18062787 PMC8002104 33801814

[13] Cardinale M Whiteley R Hosny AA Popovic N. Activity profiles, positional differences of handball players during the world championships in qatar 2015. Int J Sports Physiol Perform. (2017) 12:908–15. 10.1123/ijspp.2016-0314 27918655

[14] González-Haro P Gómez-Carmona C Bastida-Castillo A Rojas-Valverde D Gómez-López M Pino Ortega J. Analysis of playing position, match status-related differences in external load demands on amateur handball: a case study. Rev Bras de Cineantropometria e Desempenho Hum. (2020) 22:13. 10.1590/1980-0037.2020v22e71427

[15] Venzke J Schäfer R Niederer D Manchado C Platen P. Metabolic power and success in men’s handball in the European championship. In In: Dela F, Piacentini M, Helge J, Calvo Lluch Á, Sáez E, Pareja Blanco F, Tsolakidis E, editors. *27th Annual Congress of the European College of Sport Science 30 August – 2 September 2022 Book of Abstracts*, Sevilla, Spanien: European College of Sport Science (2022). 217 p.

[16] Fleureau A Rabita G Leduc C Buchheit M Lacome M. Peak locomotor intensity in elite handball players: a first insight into player position differences and training practices. J Strength Cond Res. (2023) 37(2):432–8. 10.1519/JSC.0000000000004247 36026458

[17] Michalsik LB Aagaard P Madsen K. Locomotion characteristics, match-induced impairments in physical performance in male elite team handball players. Int J Sports Med. (2013) 34:590–9. 10.1055/s-0032-1329989 23258606

[18] Póvoas SCA Ascensão AAMR Magalhães J Seabra AT Krustrup P Soares JC, et al. Analysis of fatigue development during elite male handball matches. J Strength Cond Res. (2014) 28:2640–8. 10.1519/JSC.0000000000000424 24552799

[19] Osgnach C Poser S Bernardini R Rinaldo R di Prampero PE. Energy cost, metabolic power in elite soccer: a new match analysis approach. Med Sci Sports Exerc. (2010) 42:170–8. 10.1249/MSS.0b013e3181ae5cfd 20010116

[20] Boyd LJ Ball K Aughey RJ. The reliability of minimaxx accelerometers for measuring physical activity in australian football. Int J Sports Physiol Perform. (2011) 6:311–321. 10.1123/ijspp.6.3.311 21911857

[21] Hoppe MW Baumgart C Polglaze T Freiwald J. Validity and reliability of GPS and LPS for measuring distances covered and sprint mechanical properties in team sports. PLoS ONE. (2018) 13:21. 10.1371/journal.pone.0192708 PMC5805339 29420620

[22] Alt PS Baumgart C Ueberschär O Freiwald J Hoppe MW. Validity of a local positioning system during outdoor and indoor conditions for team sports. Sensors. (2020) 20:10. 10.3390/s20205733 PMC7601858 33050174

[23] Gamble ASD Bigg JL Pignanelli C Nyman DLE Burr JF Spriet LL. Reliability and validity of an indoor local positioning system for measuring external load in ice hockey players. Eur J Sport Sci. (2023) 23(3):311–8. 10.1080/17461391.2022.2032371 35062856

[24] Blauberger P Marzilger R Lames M. Validation of player and ball tracking with a local positioning system. Sensors (Basel). (2021) 21:13. 10.3390/s21041465 PMC7923412 33672459

[25] Fleureau A Lacome M Buchheit M Couturier A Rabita G. Validity of an ultra-wideband local positioning system to assess specific movements in handball. Biol Sport. (2020) 37:351–7. 10.5114/biolsport.2020.96850 33343068 PMC7725040

[26] Luteberget LS Holme BR Spencer M. Reliability of wearable inertial measurement units to measure physical activity in team handball. Int J Sports Physiol Perform. (2018) 13:467–73. 10.1123/ijspp.2017-0036 28872371

[27] Pillitteri G Thomas E Battaglia G Navarra GA Scardina A Gammino V, et al. Validity and reliability of an inertial sensor device for specific running patterns in soccer. Sensors. (2021) 21:10. 10.3390/s21217255 PMC8587914 34770566

[28] Luteberget LS Spencer M Gilgien M. Validity of the catapult clearsky t6 local positioning system for team sports specific drills, in indoor conditions. Front Physiol. (2018) 9:10. 10.3389/fphys.2018.00115 29670530 PMC5893723

[29] Mair P Wilcox R. Robust statistical methods in R using the WRS2 package. Behav Res Methods. (2020) 52:464–88. 10.3758/s13428-019-01246-w 31152384

[30] Wilcox RR Tian TS. Measuring effect size: a robust heteroscedastic approach for two or more groups. J Appl Stat. (2011) 38:1359–68. 10.1080/02664763.2010.498507

[31] Fox J Weisberg S. An R companion to applied regression. 3rd ed. Thousand Oaks, CA: Sage (2019).

[32] R Core Team. *R: A Language and Environment for Statistical Computing*. Vienna, Austria: R Foundation for Statistical Computing (2021).

[33] Dowle M Srinivasan A. data.table: extension of ‘data.frame‘ (2022). Available from: https://r-datatable.com, https://Rdatatable.gitlab.io/data.table, https://github.com/Rdatatable/data.table.

[34] Fikenzer S Fikenzer K Laufs U Falz R Pietrek H Hepp P. Impact of COVID-19 lockdown on endurance capacity of elite handball players. J Sports Med Phys Fitness. (2021) 61:977–82. 10.23736/S0022-4707.20.11501-9 33269880

[35] Font R Irurtia A Gutierrez J Salas S Vila E Carmona G. The effects of COVID-19 lockdown on jumping performance and aerobic capacity in elite handball players. Biol Sport. (2021) 38:753–9. 10.5114/biolsport.2021.109952 34937987 PMC8670811

[36] Manchado C Tortosa Martínez J Pueo B Cortell Tormo JM Vila H Ferragut C, et al. High-performance handball player’s time-motion analysis by playing positions. Int J Environ Res Public Health. (2020) 17:15. 10.3390/ijerph17186768 PMC7559068 32957441

[37] Venzke J Schäfer R Niederer D Manchado C Platen P. Metabolic power in the men’s European Handball Championship 2020. *SportRxiv*. 10.1080/02640414.2023.2223413 37315083

[38] Thorpe RT Atkinson G Drust B Gregson W. Monitoring fatigue status in elite team-sport athletes: implications for practice. Int J Sports Physiol Perform. (2017) 12:S2-27–S2-34. 10.1123/ijspp.2016-0434 28095065

[39] Brochhagen J Ackermann N Heinrich L Hoppe M. GPS-Daten zur Detektion von muskulärer Ermüdung in den Sportspielen. LSB. (2022) 63:57–74.

[40] Fuchs PX Fuchs P von Duvillard SP Wagner H Shiang T-Y. Critical assessment of a wide-spread method for estimating energy expenditure during accelerated running based on positioning tracking systems. J Sport Health Sci. (2022) 11:641–3. 10.1016/j.jshs.2022.03.001 35263686 PMC9729919

[41] Fuchs P Luteberget LS Fuchs PX Wagner H. Comparative analysis of the indirect calorimetry and the metabolic power method to calculate energy expenditure in team handball. Appl Sci. (2022) 12:10. 10.3390/app12041952

[42] Brochhagen J Hoppe MW. Metabolic power in team and racquet sports: a systematic review with best-evidence synthesis. Sports Med Open. (2022) 8:133. 10.1186/s40798-022-00525-9 36282365 PMC9596658

[43] Javanmardi S Baumgart C Hoppe MW Freiwald J. Sprunghöhenerfassung mittels des Kinexon Tracking-Systems – eine Pilotstudie. Vol. 1 of Leipziger Sportwissenschaftliche Beiträge. Lehmanns Media (2022). p. 44–56.

